# A family with hereditary cerebellar ataxia finally confirmed as Gerstmann-Sträussler-Scheinker syndrome with P102L mutation in PRNP gene

**DOI:** 10.17712/nsj.2017.2.20160522

**Published:** 2017-04

**Authors:** Ling Long, Xiaodong Cai, Yaqing Shu, Zhengqi Lu

**Affiliations:** *From the Department of Neurology (Long, Shu, Lu), The Third Affiliated Hospital of Sun Yat-Sen University, and from the Department of Neurology (Cai), The Sixth Affiliated Hospital of Sun Yat-Sen University, Guangzhou 510655, Guangdong, People’s Republic of China*

## Abstract

Gerstmann-Sträussler-Scheinker syndrome (GSS) is an exceedingly rare prion disease. There are only 3 case reports of GSS in China. Here we report the first GSS family in southern China. A 47-year-old female complained of unsteady gait and dysarthria. Seven other individuals presented similar symptoms in 3 generations of her family, and all died 4–6 years after onset. To detect causative mutations, we employed a gene analysis panel of hereditary diseases. This revealed a P102L mutation in the prion protein gene (PRNP) gene, which is commonly found in GSS featuring cerebellar ataxia. However, GSS is an uncommon cause of hereditary cerebellar ataxia that might be overlooked because many neurologists are unfamiliar with it. To avoid misdiagnosis in the patients with hereditary cerebellar ataxia, GSS should be taken into account if other causes are absent, especially in patients that have accompanying psychiatric symptoms and a short survival time.

Cerebellar ataxia can present with many hereditary neurological diseases, including spinocerebellar ataxia, friedreich’s ataxia, episodic ataxia, and ataxia telangiectasia. In some situations, it is difficult to make a definite diagnosis based on clinical manifestations. Gerstmann-Sträussler-Scheinker syndrome (GSS) is a rare hereditary disease that can cause ataxia, although it is not well known among neurologists and is, therefore, easily misdiagnosed. We diagnosed a GSS case, who, together with her mother, presented cerebellar ataxia but were previously misdiagnosed. Our case is the fourth case report of this rare disease in China. This report briefly reviews GSS and highlights the need to consider GSS in hereditary ataxia.

## Case Report.

A 47-year-old female patient from Jiangxi Province presented with an unstable gait and dysarthria. She had been unable to walk steadily for the past 3 years. In addition, during this time, her speech became slurred. She occasionally choked when drinking water. Her symptoms gradually worsened and 1 year ago she was diagnosed with cervical disc herniation after cervical magnetic resonance imaging (MRI) at a local hospital. She underwent micro-invasive cervical vertebral surgery but did not improve. The symptoms were not episodic. She did not complain of memory loss, and she did not have any psychiatric symptoms, such as dementia, anxiety, depression, hallucinations or delusions. She had no history of frequent diarrhea, and no history of drug or food allergy. She was not an alcoholic. Exposure to toxic agents was denied. In addition, she had not taken any drugs for a long time.

**Figure 1:**
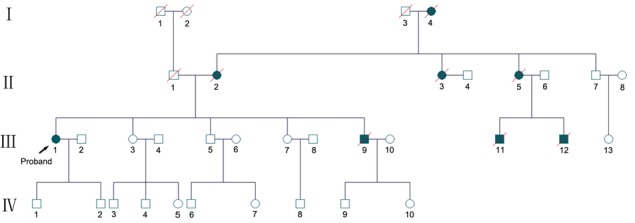
- Family tree of the pedigree. Eight members across 3 generations were involved. All have died been dead except the proband.

Her family pedigree shows evident history of ataxia (**Figure 1**), with 8 family members in 3 generations presenting ataxia. Her parents were non-consanguineous. Her mother was hospitalized in another hospital with similar symptoms at the age of 55. The diagnosis was cerebellar atrophy, but the etiology was unclear. She died when she was 59 years old. The proband’s younger brother developed symptoms of ataxia and depression at the age of 35, and committed suicide 4 years later. All of the involved members died between the ages of 35 and 59, about 4-6 years after disease onset.

Physical examination of our patient showed dysarthria, horizontal nystagmus when staring on both sides, vertical nystagmus when staring upward, wide base and unsteady gait, and difficulty with finger-to-nose test and heel-knee-shin test. Rapid alternating movements of hands was also clumsy. Romberg’s sign was abnormal with eyes open and closed. Babinski’s sign was positive for both feet and other pathological reflexes were negative. Muscle tone was mildly decreased, and muscle strength was normal without muscular atrophy. The sensation system was normal. There was no orthostatic hypotension or other autonomic nervous system dysfunction. There was no insight impairment. The Mini-Mental State Examination (MMSE) was within the normal range according to her education, showing normal calculation, language, and execution abilities, accompanied by mildly impaired short-term memory.

**Figure 2:**
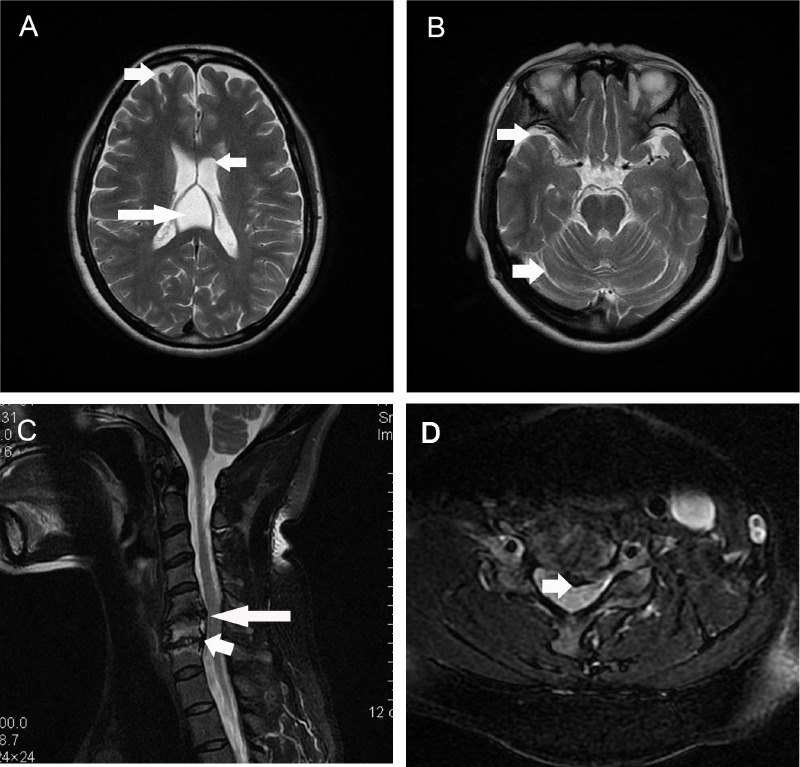
- Magnetic resonance imaging (MRI) of the brain and spinal cord. Brain MRI showed existence of cavum vergae long arrow on panel (**A**), and relatively deep sulcuses short arrow on panels (**A and B**) suggesting mild diffuse brain atrophy (**A, B**). Intervertebral herniation in cervical 5/6 (C5/6) long arrow on panels (**C and D**) and C6/7 short arrow on panel (**C**) was detected by cervical spinal cord MRI, with mild degeneration of the associated spinal cord.

Routine blood tests, such as blood cell counts and electrolytes, and routine urine and stool tests were normal. Blood vitamin (vitamin A, B1, B2, B6, B9, B12 and E) levels and serum copper concentration were within normal ranges. Thyroid function was normal. Tumor markers were negative. Antibodies for common autoimmune diseases, including autoimmune encephalitis and paraneoplastic syndromes, were negative. Additionally, electroencephalogram (EEG) and cerebrospinal fluid tests were both normal. Brain MRI indicated cavum vergae, and mild diffuse brain atrophy (**Figure 2 A, B**). Cervical spinal cord MRI revealed intervertebral herniation in cervical 5/6 (C5/6) and C6/7, leading to mild degeneration of the associated spinal cord (**Figure 2 C, D**).

**Figure 3:**
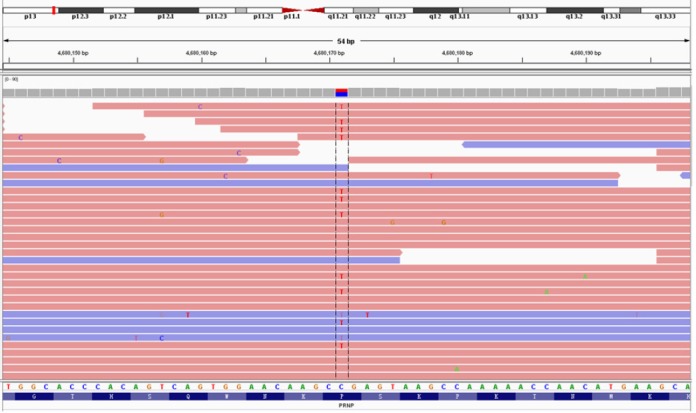
- Gene mapping of the patient. A missense mutation (C to T) was identified at nt 305 in one allele of the prion protein gene (PRNP), leading to a proline (Pro) to leucine (Leu) change at codon 102.

The brain MRI made a limited contribution to the diagnosis. Cervical intervertebral herniations were observed but they could not explain dysarthria and nystagmus. As the proband presented cerebellar ataxia and had an obvious family history, we were sure that she had hereditary cerebellar ataxia. There were no specific symptoms or signs that indicated a certain disease. To enable a definite diagnosis, we analyzed a gene panel that included 4800 genes related to common and rare hereditary diseases (designed by Kingmed Diagnostics, Guangzhou, China), including those causing spinocerebellar ataxia, Friedreich’s ataxia, Bassen-Kornzweig syndrome, Hartnup disease, Joubert’s syndrome, and ataxia with vitamin E deficiency. And also a bunch of other rare diseases. We identified a heterozygous mutation: prion protein gene (PRNP) Exon2 c.305C>T p.(Pro102Leu) (**Figure 3**), which is present in Gerstmann-Sträussler-Scheinker syndrome (GSS). Thus, the patient was eventually diagnosed as GSS. She and her family refused to do brain biopsy. Her 2 sons were not affected but did not give consent to PRNP gene analysis. We treated the patient with buspirone (it is reported to improve coordination movement in patients with ataxia,^1^ though the effect is controversial^2^) and mecobalamine (as a regular neurotrophic drug). The symptoms of limb ataxia were initially slightly relieved but then progressively worsened several weeks later. In the 14 months since we made the diagnosis, the patient informed us by telephone follow-up that she falls and chokes much more frequently and often needs help with walking. However, there is still no cognitive or behavioral abnormality.

## Discussion

Prion diseases are fatal neurodegenerative diseases of humans and animals. They include Creutzfeldt-Jakob disease (CJD), GSS, fatal familial insomnia (FFI), and Kuru. The pathogenesis of prion diseases is associated with misfolding and aggregation of the host-encoded cellular prion protein (PrP^C^). The GSS displays autosomal dominant inheritance. More than 300 cases have been reported, and the prevalence of it is 1-10/100 million inhabitants/year.^3^ Only three GSS families have been reported in China as we know (in Jilin^4^, Shanghai^5^ and Taiwan^6^). Our report is, therefore, the first in southern China. The onset age of GSS is between 30 and 60 years with a mean survival time of 3.5-9.5 years.^7^ Clinical symptoms vary within a family and between generations. Common symptoms are cerebellar ataxia, dementia, blindness, deafness, myoclonus, spastic paraparesis, parkinsonian signs, and hyporeflexia or areflexia in the lower extremities.^7^ The present family is characterized by cerebellar ataxia and only one member suffered depression. Our patient had a family history with involvement in 3 generations. All of the other affected members have died, leaving the proband as the only living family member with a confirmed diagnosis.

There are no specific laboratory or imaging criteria for diagnosing GSS. The EEG is not informative and 14-3-3 proteins are also absent in GSS. Neuropathology can be useful for diagnosing GSS. Neuropathological features of GSS present predominantly as an amyloidopathy, and other pathological changes include neuronal loss, astrocyte microgliosis and variable neurofibrillary tangles. Spongiform change is severe to absent.^7^ The most sensitive and specific way of diagnosing GSS is to detect PRNP gene mutations. GSS-related PRNP mutations include, P102L, P105L, A117V, Q160X, F198S, Q217R, Y218N, Y226X and Q227X, among which P102L is the most common.^7^,^8^ Patients with the P102L mutation are characterized with cerebellar ataxia, and can have predominant psychiatric symptoms such as apathy and depression, which is consistent with our case. P102L is considered to be the pathogenetic mutation of GSS, and experiments of Kraus et al^9^ showed that P102L expedited the spontaneous formation of amyloid in the presence of scrapie-free normal brain homogenate. It has been reported that codon 102 mutation is occasionally coupled with polymorphisms in codon 129 of PRNP,^10^ and it was MM homozygous at this location in our patient. The GSS is a fatal degenerative disease regardless of any treatments. The mean survival time is 3.5-9.5 years, and is 4-6 years in the present family. There are only 3 GSS families reported in China. The first cases were reported in 1993 in Jilin; 5 patients in 2 generations were confirmed by pathology.^4^ Another family from Shanghai (2 patients in 3 generations) was then diagnosed by pathology and gene tests (presenting the P102L mutation)^5^ and a large family from Taiwan of 4 generations and 8 patients with the P102L mutation was reported in 2009.^6^ These patients all manifested with dominant cerebellar ataxia, with or without dementia and psychiatric symptoms. The gene analysis results for the present patient were unexpected because of the low incidence of GSS. Without analyzing the gene panel containing a broad range of hereditary diseases, the diagnosis would remain ambiguous. Thus we estimate that some cases have been misdiagnosed because of insufficient knowledge of GSS.

In conclusion, GSS patients often presented with cerebellar ataxia, which mimics other hereditary diseases, such as spinocerebellar ataxia and Friedreich’s ataxia, etc. Because GSS is particularly rare, it is usually overlooked clinically. The family we present here is the fourth in China, and the first in southern China. The other involved members of this family all died before diagnosis could be confirmed. Thus we make an appeal to consider GSS in patients with hereditary cerebellar ataxia if there is no other confirmed etiology.
